# *Daphnia*’s Adaptive Molecular Responses to the Cyanobacterial Neurotoxin Anatoxin-α Are Maternally Transferred

**DOI:** 10.3390/toxins13050326

**Published:** 2021-04-30

**Authors:** Anke Schwarzenberger, Dominik Martin-Creuzburg

**Affiliations:** Limnological Institute, University Konstanz, Mainaustraße 252, 78464 Konstanz, Germany; dominik.martin-creuzburg@uni-konstanz.de

**Keywords:** cyanotoxin, maternal effects, *Daphnia* clones, *T. bourrellyi*, very fast death factor, nicotine-acetylcholin receptors

## Abstract

Cyanobacterial blooms are an omnipresent and well-known result of eutrophication and climate change in aquatic systems. Cyanobacteria produce a plethora of toxic secondary metabolites that affect humans, animals and ecosystems. Many cyanotoxins primarily affect the grazers of phytoplankton, e.g., *Daphnia*. The neurotoxin anatoxin-α has been reported world-wide; despite its potency, anatoxin-α and its effects on *Daphnia* have not been thoroughly investigated. Here, we investigated the effects of the anatoxin-α-producing *Tychonema* on life-history parameters and gene expression of nicotine-acetylcholine receptors (NAR), the direct targets of anatoxin-α, using several *D. magna* clones. We used juvenile somatic growth rates as a measure of fitness and analyzed gene expression by qPCR. Exposure to 100% *Tychonema* reduced the clones’ growth rates and caused an up-regulation of NAR gene expression. When 50% of the food consisted of *Tychonema*, none of the clones were reduced in growth and only one of them showed an increase in NAR gene expression. We demonstrate that this increased NAR gene expression can be maternally transferred and that offspring from experienced mothers show a higher growth rate when treated with 50% *Tychonema* compared with control offspring. However, the addition of further (anthropogenic) stressors might impair *Daphnia*’s adaptive responses to anatoxin-α. Especially the presence of certain pollutants (i.e., neonicotinoids), which also target NARs, might reduce *Daphnia*’s capability to cope with anatoxin-α.

## 1. Introduction

Over the last decades, cyanobacterial blooms have increased in frequency in freshwater ecosystems because of the combined effects of eutrophication, global warming, and low riverflows due to drought conditions [1]. Cyanobacteria are a risk to the environment and human health because they produce a variety of toxins [2,3]. One exceptionally dangerous toxin for humans, livestock and wildlife is the Very Fast Death Factor [4,5]—the neurotoxic alkaloid anatoxin-α. This toxin acts very quickly on the nervous system and an antidote is unknown. Anatoxin-α has been observed in natural lake blooms globally [6,7,8,9,10,11,12]. Furthermore, it is produced by different cyanobacterial species, e.g., *Tychonema* [12], *Anabaena* [7,13,14], *Pseudoanabaena*, *Planktothrix* [7] and *Aphanizomenon* [15]. Therefore, the management of cyanobacterial blooms with anatoxin-α is crucial because this toxin is a concern for human, animal and ecosystem health [6], and because anatoxin-α has already been found in dietary supplements containing cyanobacteria [16].

Anatoxin-α is probably transferred through the food-web via the ingestion of zooplankton such as *Daphnia* and affects both fish and zooplankton [8,15]. Furthermore, anatoxin-α can accumulate in the tissues of fish [8]. Representatives of the genus *Daphnia* occupy a central position in freshwater food webs. They exert a significant grazing pressure on phytoplankton, including cyanobacteria, and serve as food for zooplanktivorous fish and invertebrate predators. Especially at high temperatures, population growth rates of *D. pulex* have been shown to be negatively affected by the ingestion of an anatoxin-α-producing cyanobacterium [14]. However, anatoxin-α has been observed to have a positive effect on *D. dentifera* by preventing infection by the fungal parasite *Metschnikowia* [13].

In vertebrates, anatoxin-α inhibits the nicotine-acetylcholine receptors (NARs) [4,17,18]. This can result in the permanent stimulation of muscles, which in turn leads to paralysis and possibly death due to respiratory arrest [19]. It is not known whether *Daphnia*‘s NARs are also affected by anatoxin-α, and whether or not *Daphnia* possess the potential to adapt to anatoxin-α by specific molecular responses. Molecular adaptations and responses to other cyanobacterial toxins with other modes of action have already been observed in *Daphnia*: One example of these are microcystins, which inhibit protein phosphatases II of *Daphnia* in vitro [20]. *Daphnia* respond to this toxin type by manifold means such as: (a) Higher activity of the detoxifying enzyme gluthathion-S-transferase [21], (b) up-regulation of certain transporters, most likely in order to export microcystins from the cells [22,23,24], and (c) increased activity of malate dehydrogenase [21]. In the case of dietary protease inhibitors (PIs), *D. magna* has been demonstrated to increase protease gene expression and activity [25,26]. Furthermore, *Daphnia* populations can locally adapt to PIs [27]. This adaptation is a result of the positive selection of the respective protease type [28], production protease isoforms that are different from those produced by sensitive populations, and a change in protease gene copy number which leads to increased protease activity and higher gene expression.

Furthermore, it has been shown that if *Daphnia* mothers ingest toxic cyanobacteria, their offspring show higher fitness (i.e., higher growth or survival) than offspring from unexposed mothers [29,30,31]. Those positive maternal effects on offspring fitness were shown to be due to the transfer of the specific molecular responses from mothers to their offspring (microcystins: higher activity of glutathione S-transferase and malate dehydrogenase [21]; protease inhibitors: increase in protease gene expression [30]). In the case of anatoxin-α, it is not known whether similar maternal effects can increase offspring fitness in *Daphnia*, and whether a putative specific response, e.g., increased NAR gene expression, is maternally transferred.

The aim of this study was to investigate the effects of anatoxin-α on *D. magna* and the capability of *D. magna* to respond and adapt to this toxin type by increasing NAR gene expression. For this, we measured the juvenile somatic growth rates of three different *D. magna* clones grown on different concentrations of the anatoxin-α-producing cyanobacterium *T. bourrellyi* and on a control food (the green alga *Scenedesmus obliquus*), we also quantified their NAR gene expression. Furthermore, we treated the F0 generation of one *D. magna* clone with two different concentrations of *T. bourrellyi* and measured the maternal and offspring gene expression and fitness of the exposed and naïve F1 offspring in maternal effect experiments.

## 2. Results

### 2.1. Clones

#### 2.1.1. Somatic Growth Rates of the Three *D. magna* Clones

**Figure 1:**
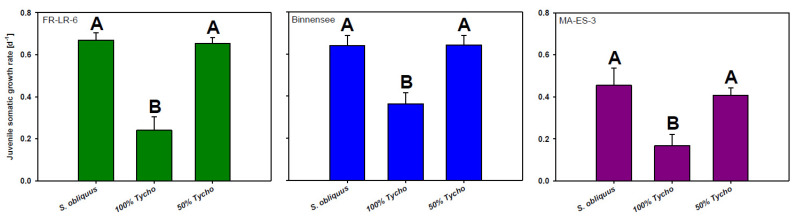
Juvenile somatic growth rates (mean + SD) of three *D. magna* clones (‘Binnensee’, ‘MA-ES-3’, ‘FR-LR6-1’) grown either on 100% *S. obliquus*, 100% *T. bourrellyi* (Tycho) or a mixture of 50% *S. obliquus* with 50% *T. bourrellyi* for four days. Different letters indicate significant differences between treatments in a single clone (*p* < 0.05).

The clones achieved similar growth rates both on the control food (100% *S. obliquus*) and on the mixed diet containing 50% of *T. bourrellyi*; growth rates were significantly reduced only on the 100% *T. bourrellyi* diet (Tukey’s HSD after one-way ANOVA: FR-LR-6-1: F_2,6_ = 1175.2; *p* < 0.001; Binnensee: F_2,6_ = 1116.7; *p* < 0.001; MA-ES-3: F_2,6_ = 291.5; *p* = 0.002; Figure 1). The culture’s anatoxin-α concentration in the experiments was 161.8 ng per mg carbon.

#### 2.1.2. Gene Expression

**Figure 2:**
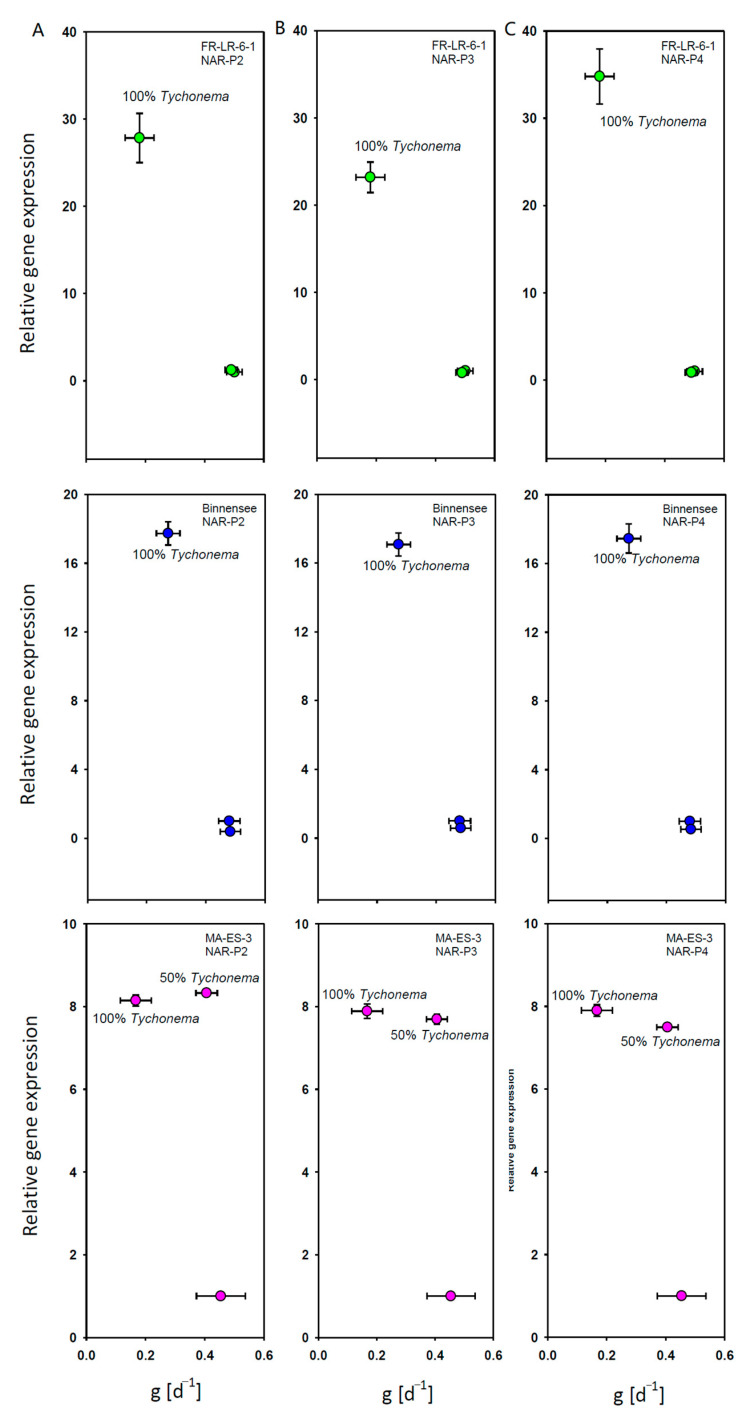
Relative expression of three nicotine-acetylcholine receptor genes (**A**) NAR-P2, (**B**) NAR-P3, and (**C**) NAR-P4 as a function of juvenile somatic growth rates (g [d^−1^]) of the three *D. magna* clones when grown on 100% *S. obliquus*, 100% *T. bourrellyi* or a mixture of 50% *S. obliquus* with 50% *T. bourrellyi*.

In the clones FR-LR6-1 and Binnensee, both the gene expression of the three nicotine-acetylcholine receptor genes and the juvenile somatic growth rates were similar between the control and 50% *T. bourrellyi*. In the treatment with 100% *T. bourrellyi*, an increase in gene expression was accompanied by a lower somatic growth rate (Figure 2A,B). In contrast, somatic growth in clone MA-ES-3 was similar on the control food and 50% *T. bourrellyi*, but the gene expression already increased on 50% *T. bourrellyi* (Figure 2C). 

### 2.2. Maternal Effects

#### 2.2.1. First Experiment: 50% *T. bourrellyi*

**Figure 3:**
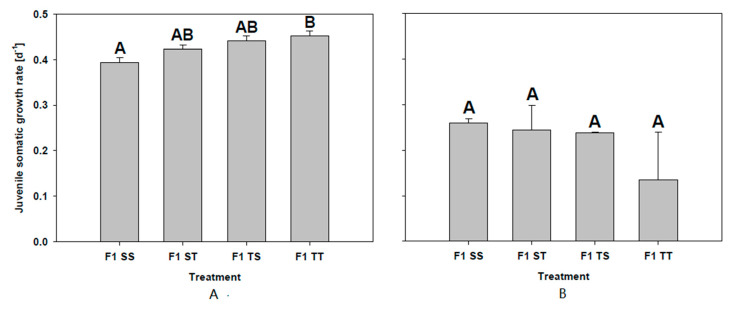
Juvenile somatic growth rates (mean + SD) of the F1 generation of the maternal effect experiments (clone MA-ES-3) with either 50% *S. obliquus* and 50% *T. bourrellyi* (**A**) or 100% *T. bourrellyi* (**B**) as cyanobacterial maternal and offspring food treatment. Different letters indicate significant differences between offspring treatments (*p* < 0.05).

No difference in the juvenile somatic growth rates was observed between animals grown on the control food or on 50% *T. bourrellyi*. However, a significantly higher growth rate was found in F1 TT in comparison to F1 SS (Tukey’s HSD after one-way ANOVA F_3,8_ = 8320.70; *p* = 0.015; Figure 3A). Neither clutch size nor age at first reproduction differed between the treatments of the two F1 groups (clutch size; **F1SS:** 14.33 ± 1.15; **F1ST:** 20 ± 7.81; **F1TS:** 14.66 ± 1.15; **F1TT:** 16.67 ± 7.37; one-way ANOVA F_3,8_ = 901.04; *p* = 0.726; age at first reproduction: **F1SS:** 10 ± 0; **F1ST:** 9.67 ± 0.58; **F1TS:** 10 ± 0; **F1TT:** 9.33 ± 1.16); one-way ANOVA F_3,8_ = 12241.42; *p* = 0.556).

**Figure 4:**
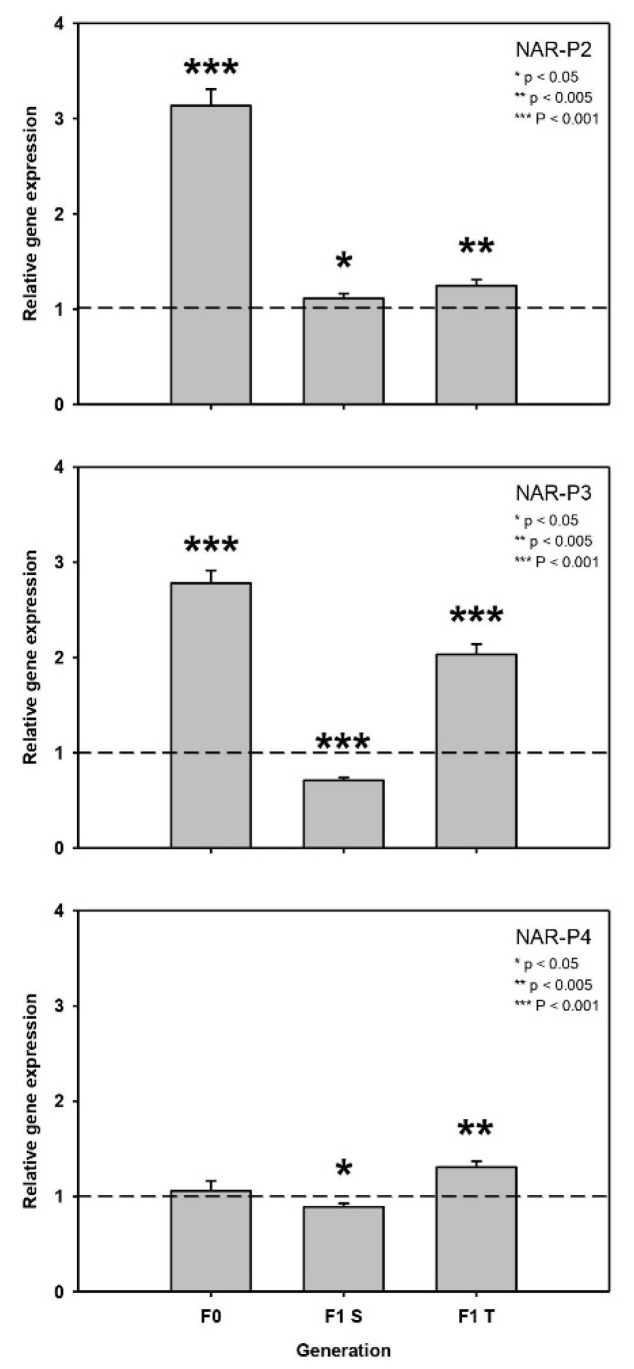
Relative expression (mean + SD) of three nicotine-acetylcholine receptor genes (NAR-P2, NAR-P3, and NAR-P4) of the F0 and F1 generations from the first maternal effect experiment (clone MA-ES-3 with 50% *S. obliquus* and 50% *T. bourrellyi* as maternal and offspring food treatment). Asterisks indicate significant differences (*t*-tests, *p* < 0.05) between control and 50% *T. bourellyi*. Gene expression on control food served as a calibrator and was always set to 1 (dashed line in the graphs).

The genes NAR-P2 and NAR-P3 were significantly up-regulated on *T. bourellyi*, both in the F0 and F1 T generations (after six days), but Nar-P2 was up-regulated and Nar-P3 down-regulated in F1 S (**NAR-P2: F0:** t = 16.44, *p* < 0.001; **F1S:** t = 3.02, *p* = 0.039; **F1T:** t = 6.67, *p* = 0.003; **NAR-P3: F0:** t = 21.62, *p* < 0.001; **F1 S:** t = -11.87, *p* < 0.001; **F1 T:** t = 16.77, *p* < 0.001; Figure 4). NAR-P4 was not regulated in F0, was slightly down-regulated in F1 S and was up-regulated in F1 T (**NAR-P4: F0:** t = 0.85, *p* = 0.445; **F1 S:** t = -3.97, *p* = 0.017; **F1 T:** t = 8.298, *p* = 0.001; Figure 4).

#### 2.2.2. Second Experiment: 100% *T. bourrellyi*

Because of the high mortality in F1 ST and F1 TT (**F1SS:** 0; **F1ST:** 73.33 ± 11.55%; **F1TS:** 0; **F1TT:** 90 ± 52.92%), the experiment was terminated on day four and the juvenile somatic growth rates were compared; no difference was found between the treatments and the offspring groups (one-way ANOVA F_3,7_ = 64.94; *p* = 0.174; Figure 3B).

## 3. Discussion

Anatoxin-α has been shown to induce behavioural changes, inhibit heart rate and alter oxygen consumption in *D. magna* [32]. Claska and Gilbert [3] reported reduced fecundity of *D. pulex* after the ingestion of an anatoxin-α-producing cyanobacterium. Furthermore, the growth rates and survival of *D. similis* were negatively affected [33]. Similarly, our tested *D. magna* clones which had ingested *T. bourellyi* were strongly and significantly reduced in growth when fed with 100% but not with 50% of the cyanobacterium. Although other unknown cyanotoxins might be present in *T. bourellyi* and might affect *Daphnia*, we feel confident that the negative effect on juvenile somatic growth rates was likely because of the presence of anatoxin-α. This is because (i) our *T. bourellyi* strain does not produce microcystins [12], and (ii) when we fed another *D. magna* clone with an aging *T. bourellyi* culture in which anatoxin-α was not detected via ELISA, we found no growth rate reduction on 100% *T. bourellyi* (Supplementary Figure S1).

It has been demonstrated that organisms can respond to anatoxin-α exposure by increasing the activity of detoxifying enzymes sucj as peroxidases and glutathione S-transferases [34]. However, until now it was unclear whether organisms can also respond or even adapt to anatoxin-α by changes in the production of the direct targets of anatoxin-α, i.e., NARs [16,17,35]. It has been shown that *D. magna* can respond to other cyanotoxins by increasing the gene expression and activity of the cyanotoxins’ direct targets (cf. protease inhibitors and dietary proteases [26]). We found that the gene expression of three NARs increased when the three *D. magna* clones were fed with 100% *T. bourellyi*. In MA-ES-3, this increase in gene expression was already observed on 50% *T. bourellyi*, which was accompanied by a similar growth rate as on the control food. This suggests that *D. magna* can actually respond and probably adapt to anatoxin-α by increasing target gene expression.

It is advantageous for parents and offspring to show the same phenotype if they share the same risk of exposure to the same deleterious environment. Adaptive phenotypic responses can be maternally transferred to the offspring, affecting the offspring’s development and ultimately its fitness [36]. A maternal effect is adaptive if the offspring’s fitness increases. The generation time of *Daphnia* is typically shorter than the exposure to cyanobacteria. Therefore, the maternal transfer of a certain phenotype is beneficial. For cyanobacterial protease inhibitors it has been shown that the mothers’ (F0 generation) increase in gene expression was also transferred to their offspring (F1 generation), and that this transfer of gene expression was accompanied by a higher fitness of the offspring of experienced mothers [30]. We hypothesized that such a maternal effect could also be found in clone MA-ES-3 in response to anatoxin-α. In fact, we found that in the experiment with 50% *T. bourellyi* the higher gene expression of NAR-P2 and NAR-P3 in the experienced mothers was also observed as increased gene expression in F1 TT in comparison to F1 TS. A higher gene expression of NAR-P2 was also observed in the offspring of naïve mothers, i.e., F1 ST in comparison to F1 SS; however this increase was clearly lower than in the offspring of experienced mothers. Furthermore, a gene expression increase of NAR-P4 was observed in F1 TT in comparison to siblings grown on the control food, although the increase was neither observed in their experienced mothers nor in the offspring of naïve mothers (F1 ST). Although the growth rate, clutch size, and age until the first reproduction of the offspring of both naïve and experienced mothers was not different on thecontrol food, we found that the F1 TT offspring of experienced mothers showed a higher growth rate than the F1 SS offspring of naïve mothers. Presumably, the effects of anatoxin-α on the growth of the F1 TT offspring were suppressed by the increase in NAR gene expression. Furthermore, it is possible that with this elimination of toxin stress, the offspring benefitted from the mixed diet by showing an even higher growth than the F1 SS offspring. This seems likely, since nutritional upgrading by non-toxic bacteria has already been described [37]. We concluded that the maternal transfer of increased gene expression led to a higher fitness in the F1 generation of experienced mothers.

We wanted to test whether this effect was more apparent when the offspring was stressed with even higher concentrations of *T. bourellyi* (100%). The experienced mothers were still grown on 50% *T. bourellyi* in order to ensure a similar maternal transfer of increased gene expression as in the first experiment. We found that the juvenile somatic growth rates of all types of offspring were similar. However, the experiment was ended already after four days because the mortality of the offspring grown on *T. bourellyi* was very high (both in F1 ST and F1 TT). Therefore, no fitness effect of the maternal transfer of increased gene expression was observed in the experiment with 100% *T. bourellyi*. 

In conclusion, increased NAR gene expression is maternally transferred and leads to higher fitness in the offspring of experienced mothers if the percentage of *T. bourellyi* is not too high. It remains to be tested whether the presence of several other cyanotoxins within a bloom or the presence of additional stressors (e.g., anthropogenic stressors such as pharmaceuticals or light pollution) can reduce the positive effect of this maternal transfer. Particularly the presence of pollutants that also affect nicotine-acetylcholine receptors of arthropods (e.g., neonicotinoids [38]) might reduce *Daphnia*’s capability to cope with anatoxin-α.

## 4. Materials and Methods

### 4.1. Cultures

Experiments were conducted with an anatoxin-α producing strain of *T. bourrellyi* which was isolated from Lake Mjøsa, Norway [10]. *T. bourrellyi* was grown at 20 °C in 150 mL Cyano medium [39] at constant illumination (100 μmol quanta m^−2^ s^−1^) for seven days prior to the experiments. As the concentration of anatoxin-α decreases in aging cultures of *T. bourrellyi*, we always verified that anatoxin-α was present in the culture in two technical duplicates at day four of the experiments. For each replicate, 6 mL of the culture was centrifuged, and the pellet was resuspended with 500 µL Cyano medium and mixed with 500 µL 60% ethanol. The mix was diluted 1:100 with Millipore water, and its anatoxin-α content was determined with an ELISA kit (Anatoxin- α (VFDF) RBA, 96-test; Sension, Augsburg, Germany) as according to the manufacturer’s instructions and by making use of the calibration curve from the kit. Pure Cyano medium without *T. bourellyi* served as blank and was treated in the same way. The anatoxin-α content (in ng) was related to the carbon (C) concentration of the tested *T. bourellyi* culture via a previously determined carbon-light extinction regression. For this, one milliliter per each of six *T. bourellyi* dilutions was filtrated on a glass-fiber filter and dried at 50 °C for 24 h. Subsequently the carbon content (in mg C) of each filter was measured with a C/N analyzer. The extinction of each dilution at 470 nm was plotted against its carbon concentration and a linear regression line was drawn. From this regression line, the anatoxin-α concentration of each culture was calculated in relation to its carbon content (ng anatoxin-α per mg C).

The single-cell green alga *Scenedesmus obliquus* (SAG 276-3a, Culture Collection of Algae, University of Göttingen, Göttingen, Germany) was used as a control food. *S. obliquus* was cultured semi-continuously (dilution rate: 0.2 d^−1^) in Cyano medium at constant illumination in aerated 5 L flasks. Food suspensions were prepared by centrifugation and resuspension in fresh medium. The carbon concentrations of the *S. obliquus* and *T. bourrellyi* food suspensions were estimated from carbon–light extinction regressions established prior to the experiment.

Three *Daphnia magna* clones (‘Binnensee’ (isolated in Germany [40]), ‘MA-ES-3’ (isolated in Morocco; courtesy of D. Ebert, University of Basel, Basel, Switzerland) and ‘FR-LR6-1’ (isolated in France; courtesy of D. Ebert, University of Basel, Basel, Switzerland) were used in the experiments. They were cultivated at a day-night cycle of 16:8 h at 20 °C for several generations before the actual experiments were started: Seven individuals were kept in jars containing 250 mL filtered (<0.2 μm) and aerated Lake Constance water and saturating amounts of the green alga *S. obliquus* as food. The animals were fed daily and transferred into fresh medium every other day.

### 4.2. Experiment Set-Ups

#### 4.2.1. Clone Experiments

Ten newborns per *D. magna* clone were used for the determination of dry mass at the start of the experiments. Seven individuals per individual *D. magna* clones were grown in 200 mL filtrated and aerated Lake Constance water on 2 mg C L^−1^ of either 100% *S. obliquus*, 100% *T. bourrellyi*, or 50% *S. obliquus* and 50% *T. bourrellyi* for four days at 20 °C at a day-night cycle of 16:8 h. The experiment was run in biological triplicates. At the end of the experiment, five animals per replicate were used for dry mass determination; the remaining two were stored at −80 °C for subsequent RNA extraction. The juvenile somatic growth rates were calculated by making use of the dry mass at the start and at the end of the experiments as according to Schwarzenberger et al. (2020) [24].

#### 4.2.2. Maternal Effect Experiments

**Figure 5:**
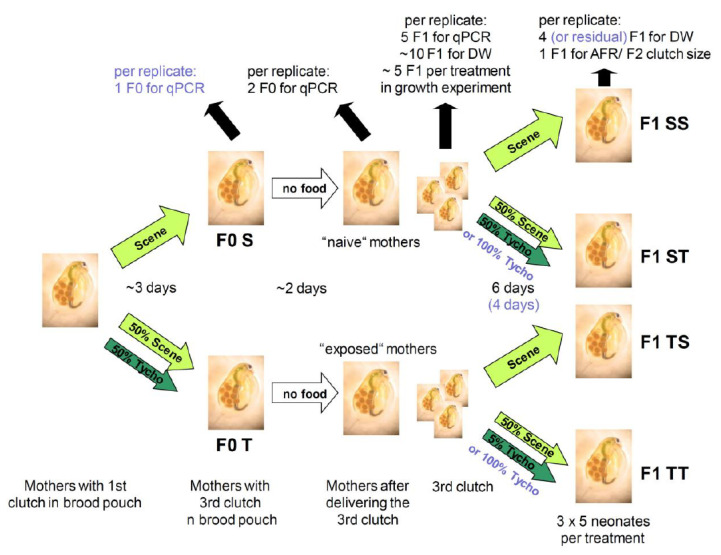
Experiment set-ups for the two maternal effect experiments (black: first experiment with 50% *T. bourrellyi*; blue in brackets: second experiment with 100% *T. bourrellyi*). T/Tycho = *T. bourrellyi*, S/Scene = *S. obliquus*, DW = dry weight, AFR = age at first reproduction.

Two maternal effect experiments were conducted according to the following set-up (Figure 5): Seven newborns of clone MA-ES-3 per six 250 mL glasses were fed *S. obliquus ad libitum* until the eggs of the first clutch were deposited into their brood pouches. The medium was exchanged every other day. Subsequently, three of the replicates were reared on 2 mg C L^−1^ of 100% *S. obliquus*, whereas the other three replicates were fed 50% *S. obliquus* and 50% *T. bourellyi* until the second clutch was released. In the second experiment, at this point in time one F0 mother per replicate was frozen previous to RNA extraction. The two different F0 generations (F0S: naïve and F0T: experienced mothers) with the third clutch eggs deposited into the brood pouches were then put into a food-free medium. Thus, we ensured that the developing juveniles in the brood pouches had never ingested any food and that all effects we expected to find were exclusively due to maternal transfer. When the third clutch was released, two F0 individuals (first experiment only) and five F1 neonates per replicate per treatment were frozen for subsequent RNA extraction. Furthermore, ten F1 neonates per replicate were dried for the determination of the start weight for the growth rates. In the subsequent growth experiment, five F1 neonates per replicate were grown on 2 mg C L^−1^ of either 100% *S. obliquus*, or 50% *S. obliquus* and 50% *T. bourellyi* (second experiment: 100% *T. bourellyi*) for six days (second experiment: four days) in 100 mL glasses. Four of the F1 neonates (second experiment: all surviving neonates) were dried for the end weight of the growth rates. One individual per replicate was cultivated until its first clutch was released, and the number of the F2 offspring was counted. Not enough individuals survived for the determination of F2 clutch size in the second experiment.

### 4.3. Gene Expression

For RNA extraction, we used the NucleoSpin^®^ RNA Kit (Macherey-Nagel) as according to the manufacturer’s instructions. The RNA concentrations and integrities were determined with the Thermo Scientific™ NanoDrop™ 2000 Spectrometer (ThermoFisher, Waltham, MA, USA). RNA was reverse transcribed with the High-Capacity cDNA Reverse Transcription Kit (Applied Biosystems™). Following the protocol of Schwarzenberger et al. (2009) [41], qPCR was performed with the 7500 Fast and 7500 Real-Time PCR System (Applied Biosystems™). Each reaction contained 5 ng cDNA (10 ng of RNA equivalent) and 1 μL of a primer pair (either *cyclophilin, ubc* [42], *NAR-P2, NAR-P3, or NAR-P3* (Table 1). From a set of ten primers, *cyclophilin* and *ubc* were chosen as controls as they proved to be the most stable between the treatments. The results were analyzed with the 7500 Software v2.3 (Applied Biosystems™). Gene expression on 100% *S. obliquus* served as a calibrator and was always set to 1. 

### 4.4. Statistics

The relative gene expression of the single-clone experiments, juvenile somatic growth rates, clutch size and age at first reproduction were analysed via one-way ANOVA and Tukey’s HSD post-hoc tests after verifying homogenous variances. When necessary, data were transformed before the analyses (log2: clutch size and age at first reproduction in the first maternal effects experiment; x^2^: juvenile somatic growth rates in the second maternal effects experiment). The relative gene expressions on *S. obliquus* and *T. bourellyi* were compared using *t*-tests in the maternal effects experiments. ANOVAs and *t*-tests were calculated with the program STATISTICA (StatSoft, Inc. 2011, version 10.0, Tulsa, OK, USA). 

## Figures and Tables

**Table 1:** Primer names, gene names (according to wfleabase.org), primer sequences, amplicon length, and gene accession numbers (acc #; wfleabase.org) of three nicotine-acetylcholine receptor genes. Their melting temperature was always 60 °C.

Primer Name	Gene Name	Primer Forward (5′–3′)	Primer Reverse (5′–3′)	Length	acc #
NAR-P2	Neuronal nicotinic acetyl-choline subunit	GGCGCAGACCTCTCTTCTAC	GGTTGTGATGCCGAGAGTCA	125 bp	JGI_V11_55424
Nar-P3	Neuronal nicotinic acetylcholine subunit	ATTTGCTTGGTGTCGTTCGC	AGAATGATCCGGCGCATGAT	107 bp	JGI_V11_66098
NAR-P4	Nicotinic acetylcholine receptor subunit beta 1	CACAACCACACGAAACCCAC	GAAGACCAAGACGCACAGGA	129 bp	JGI_V11_321681

## Data Availability

All data will be provided by the authors upon request.

## Supplementary Materials

The following are available online at https://www.mdpi.com/article/10.3390/toxins13050326/s1, Figure S1: Juvenile somatic growth rate.
