# Supplementary material for: Daphnia’s Adaptive Molecular Responses to the Cyanobacterial Neurotoxin Anatoxin-α Are Maternally Transferred
Source: Toxins (Basel). 2021 Apr 30;13(5):326. doi: 10.3390/toxins13050326 (PMC8147185; doi:10.3390/toxins13050326)
Supplement: Supplementary file 1 [file toxins-13-00326-s001.zip › toxins-1186400-supplementary.pdf]

Supplementary Materials: Daphnia's Adaptive Molecular Responses to the Cyanobacterial Neurotoxin Anatoxin-A are Maternally Transferred

Anke Schwarzenberger and Dominik Martin-Creuzburg

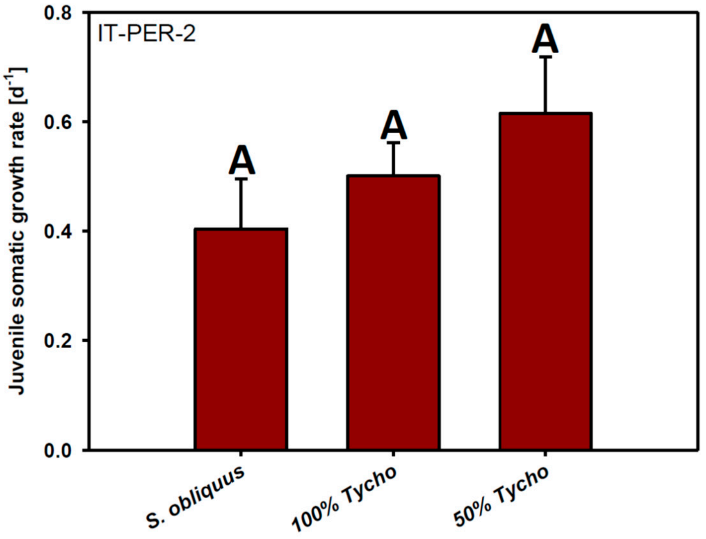

Figure S1. Juvenile somatic growth rate (mean + SD) of *D. magna* clone 'IT-PER-2' grown either on 100% *S. obliquus*, 100% *T. bourrellyi (Tycho)* or a mixture of 50% *S. obliquus* with 50% *T. bourrellyi* for four days. IT-PER-2 did not differ in growth rate between treatments (Tukey's HSD after one-way ANOVA $F_{2,5}$ = 284.61; p = 0.097). In the course of the growth experiment with IT-PER-2 we did not detect any anatoxin-α in the *T. bourellyi* culture.
